# Real‐World Evidence Assessment of the Risk of Nonfatal Stroke in Patients Prescribed SGLT2 Inhibitors

**DOI:** 10.1155/srat/6411721

**Published:** 2026-06-25

**Authors:** Alessandra T. Ayers, Cindy N. Ho, Umesh Masharani, William C. Pike, Honor Magon, Michael L. Jackson, Saurabh Gombar, Agatha F. Scheideman, Mandy M. Shao, David C. Klonoff

**Affiliations:** ^1^ Diabetes Technology Society, Burlingame, California, USA; ^2^ University of California San Francisco, San Francisco, California, USA, ucsf.edu; ^3^ Atropos Health, New York, New York, USA; ^4^ Stanford University, Stanford School of Medicine, Stanford, California, USA, stanford.edu; ^5^ Diabetes Research Institute, Mills-Peninsula Medical Center (Sutter Health), San Mateo, California, USA, diabetesresearch.org

**Keywords:** cardiovascular, hemorrhagic stroke, ischemic stroke, SGLT2 inhibitors, Type 2 diabetes

## Abstract

**Aims:**

Use of sodium‐glucose cotransporter 2 inhibitors (SGLT2i) in adults with Type 2 diabetes (AwT2D) has recently been associated with decreased stroke risk. In this study, we investigate the impact of SGLT2i on stroke.

**Materials and Methods:**

We conducted a cohort study using an electronic health record (EHR) representing 66 million patients to examine whether AwT2D have a lower incidence of stroke and/or transient ischemic attack (TIA) if treated with SGLT2i. All analyses were completed utilizing high‐dimensional propensity score matching to control for confounders.

**Results:**

AwT2D treated with SGLT2i compared to other antidiabetic medications, assessed after 1 year, starting 2 weeks after the date of prescription of SGLT2i or other antidiabetic drug, had a significantly lower incidence of any stroke (Odds Ratio (OR): 0.84 with 95% Confidence Interval (CI) 0.79–0.91, *p* < 0.001), hemorrhagic stroke (OR: 0.72 with 95% CI 0.59–0.88, *p* < 0.001), ischemic stroke (OR: 0.86 with 95% CI 0.77–0.95, *p* = 0.005), and TIA (OR: 0.87 with 95% CI 0.80–0.95, *p* = 0.003).

**Conclusion:**

Among AwT2D, those treated with SGLT2i had lower incidence of stroke and TIA compared with adults treated with other antidiabetic medications.

## 1. Introduction

In this study, we analyze whether sodium‐glucose cotransporter 2 inhibitors (SGLT2i) provide a protective effect against stroke. SGLT2i are antihyperglycemic agents used to treat adults with Type 2 diabetes (AwT2D) [1, 2]. SGLT2i inhibit sodium‐glucose cotransporter 2 (SGLT2) in the proximal tubule of the renal cortex. This results in glycosuria, lower serum glucose levels, and subsequently lower hemoglobin A1c levels over time. This class of agents has also been shown to reduce the risk of major adverse cardiovascular (CV) events [3], and especially admissions for heart failure and death from heart failure. SGLT2i also have renoprotective effects, as they reduce the risk for progression of renal disease in people with and without diabetes [4].

In the medical literature, for people with diabetes, the reported relationship of the effect of SGLT2i, compared to other drugs for diabetes, on stroke risk has been inconsistent. Most [5–7], but not every [8], review articles of the effect of SGLT2i on stroke risk have reported no overall improvement with this class of drugs. However, a recent review of this same putative relationship by Kim et al. [9] that also included patients using the sodium‐glucose cotransporter 1 (SGLT1‐) and SGLT2‐coinhibitor, sotagliflozin, noted an overall improvement in stroke risk [10]. Tsai et al. [11] and Pasqualotto et al. [5] have reported in meta‐analyses that SGLT2i have a neutral effect on the overall risk of stroke and its subtypes (all strokes and ischemic strokes as well as transient ischemic attacks), but a potential protective effect against hemorrhagic stroke [11]. The dual SGLT2/SGLT1 inhibitor sotagliflozin has been shown to reduce the risk of stroke [12].

Although the exact mechanism for the impact of SGLT2i on CV health, beyond improving blood glucose control, is unknown, there are multiple hypotheses for why SGLT2i may improve CV health, including by lowering blood pressure, altering Na^+^ balance in cardiomyocytes, promoting ketone production, inhibiting SGLT1, reducing inflammation, and interacting with the renin–angiotensin–aldosterone system [13].

In this study, we used real‐world evidence (RWE) to assess potential protective effects of SGLT2i on stroke risk. We compared those effects with the clinical relationships reported by Rodriguez‐Valadez et al. [7] from clinical trial data.

## 2. Methods

In this cohort study, we assessed the incidence of stroke among adult patients with Type 2 diabetes (T2D) mellitus aged 18–90 years in the in the Apollo dataset, as part of Atropos Health′s Evidence Network. We compared an intervention group (those treated with SGLT2i) to a control group (those prescribed any other antidiabetic agents excluding SGLT2i). We did not address whether the Coronavirus Disease of 2019 (COVID‐19) pandemic affected the incidence of stroke in this study.

We performed a retrospective real‐world cohort analysis of adult patients with T2D using SGLT2i for the risk of stroke. The dataset is a nationally representative electronic health record (EHR) and claims dataset that includes records for 66 million unique patients encompassing the years of 2015–2023. EHR data were mapped to the Observational Medical Outcomes Partnership (OMOP) Common Data Model (CDM) Version 5.3, which allowed cohort building via standardized vocabularies like the Anatomical Therapeutic Chemical (ATC) classification system, RxNorm, International Classification of Diseases 9th or 10th Revision Code (ICD‐9, ICD‐10), and Logical Observation Identifiers Names and Codes (LOINC).

### 2.1. T2D Cohort

For entry into the cohort, we required patients to have a T2D diagnosis code (as defined by an ICD‐9 or ICD‐10 code) between January 1, 2015, and December 1, 2022, followed by a second T2D ICD‐9 or ICD‐10 code between 12 and 18 months later. The use of two temporally separated codes restricted the population to patients with significant longitudinal records in the database. The later T2D ICD code was defined as the reference T2D diagnosis.

### 2.2. Study Arms

We required patients in the intervention group to have a new prescription of an SGLT2i within 30 days after their reference T2D diagnosis. We specified that members of the control cohort were required to have a prescription for any non‐SGLT2i antidiabetic drug within 30 days post‐reference T2D diagnosis code. The date of the first prescription (SGLT2i or other antidiabetic drug) within the 30‐day window after reference T2D diagnosis is denoted as the index date.

Patients were required to have a minimum follow‐up of one year after the index date (i.e., all patients must have had further records occurring at one year after the index date). Anyone who had a fatal stroke within the year post‐SGLT2i/other antidiabetic would be excluded. Patients were excluded if they had an ICD‐10 code stroke (hemorrhagic, ischemic, TIA) any time prior to the index date.

### 2.3. Outcomes

**Figure 1:**
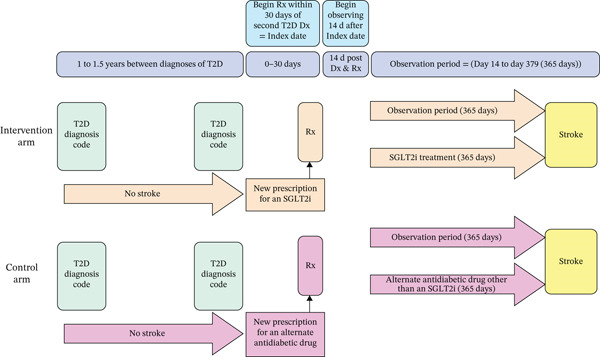
Protocol timeline. Timeline for the intervention arm and control arm for the comparison of outcomes between users of SGLT2i and users of other antidiabetic drugs. Abbreviations: d, days; Dx, diagnosis; Rx, prescription; SGLT2i, sodium‐glucose cotransporter 2 inhibitor, T2D, Type 2 diabetes.

Patients were followed for one year, starting two weeks after the index date (date of prescription of SGLT2i or other antidiabetic drug) for incidence of any stroke (inclusive of ischemic stroke, hemorrhagic stroke, and/or transient ischemic attack [TIA]), further stratified into their specific stroke groups (ischemic stroke, hemorrhagic stroke, and TIA). The window of observation for stroke was from the index date + 14 days up to the index date + 379 days in order to account for two weeks of SGLT2i/other antidiabetic usage prior to the observation window. The clinical trial timeline for the intervention arm and control arm for the comparison of outcomes between users of SGLT2i and users of other antidiabetic drugs is presented in Figure 1.

All hemoglobin or hematocrit measurements were taken in the first seven days after a stroke. Incidence of stroke was compared using a chi‐square test. Furthermore, all analyses were completed utilizing high‐dimensional propensity score (hdPS) matching (PSM) [14] to control for confounders covering demographics, clinical comorbidities, treatment year, prior procedures, medications, and healthcare utilization that each patient had prior to their index date.

To calculate the hdPS, we first extracted covariates from medical codes (ICD, Current Procedural Terminology [CPT]) and drug codes (RxNorm) [14]. We then fit these covariates into a logistic regression model using Least Absolute Shrinkage and Selection Operator (LASSO) regularization to produce equivalent predictive performance without overfitting too many covariates in a high‐dimensional setting [15, 16]. The LASSO hyperparameter is tuned using 5‐fold cross‐validation and the 1‐standard‐error rule [17].

A two‐sided *p* value of 0.05 was considered statistically significant. Furthermore, for each association, we calculated an *E*‐value which indicates how much an unmeasured confounder would need to influence both the treatment and the outcome to change the estimated effect to a null value or no effect [18]. Analyses were performed using R Version 4.2 on the Atropos Health platform.

## 3. Results

We studied an intervention group of *n* = 141,699 and an equal sized control group of *n* = 141,699. Demographic characteristics of the SGLT2i intervention group and the control group are presented in Table 1. These characteristics were measured before PSM was performed. The majority of patients in both the intervention and control groups were White (56.3% and 54%, respectively), and the control arm had a slightly higher frequency of female patients (51.9% vs. 45.4%) The mean ages of patients in the control group and intervention group were 63.2 and 61 years, respectively. The intervention group received SGLT2i, and the control group received other antidiabetic drugs. We found that the control group had a lower percentage of patients with diabetes complications (46.05%) compared to the intervention group (53.62%).

**Table 1:** Demographic characteristics of the intervention group and the control group.

	Intervention group (SGLT2 inhibitors)	Control group (other antidiabetic drugs)
Number of individuals	141,699	141,699
Female (%)	45.40%	51.90%
White (%)	56.30%	54%
Black (%)	8.90%	9.20%
Asian (%)	4.10%	3.60%
Other or unknown (%)	30.70%	33.20%
Hispanic (%)	10%	7.50%
Average age in years (SD)	61 (11.7)	63.2 (12.7)
Diabetes with complications (%)	53.62%	46.05%

*Note:* Key demographic characteristics of the intervention group, prescribed SGLT2i, versus the control group, prescribed antidiabetic drugs other than SGLT2i inhibitors.

Abbreviations: SD, standard deviation; SGLT2, sodium‐glucose cotransporter 2.

The results indicated a statistically significant lower odds ratio (OR) for the incidence of any stroke (OR: 0.84 with 95% confidence interval (CI) 0.79–0.91, *p* < 0.001), hemorrhagic stroke (OR: 0.72 with 95% CI 0.59–0.88, *p* < 0.001), ischemic stroke (OR:0.86 with 95% CI 0.77–0.95, *p* < 0.001), and TIA (OR: 0.87 with 95% CI 0.80–0.95, *p* < 0.001) in adults with T2D taking SGLT2i compared to those treated with other antidiabetic medications, from 14 to 379 days after the index event. This event was specified to be the date of prescription of SGLT2i or other antidiabetic drug,

The comparisons were constructed using PSM, with *n* = 141,699 in the intervention group and *n* = 141,699 in the control group. The <any stroke>, <hemorrhagic stroke>, <ischemic stroke>, and <TIA> outcomes had *E*‐values of 1.4, 1.5, 1.3, and 1.3, respectively. Results, as ORs, *p* values, and *E*‐values of strokes for users of SGLT2i compared to users of other antidiabetic drugs, are presented in Table 2

**Table 2:** Odds ratios of strokes for users of SGLT2i compared to users of other antidiabetic drugs.

Type of stroke	Odds ratio (SGLT2i)	*p*	*E*‐value
Any stroke	0.84	*p* < 0.001	1.4
Hemorrhagic	0.72	*p* < 0.001	1.5
Ischemic	0.86	*p* = 0.005	1.3
TIA	0.87	*p* = 0.003	1.3

*Note:* Odds ratios for patients on SGLT2i4 versus any other antidiabetic drug experiencing any stroke (hemorrhagic, ischemic, or TIA), hemorrhagic stroke, ischemic stroke, and TIA.

Abbreviations: SGLT2i, sodium‐glucose cotransporter 2 inhibitors; TIA, transient ischemic attack.

## 4. Discussion

In this analysis, using RWE, we investigated whether SGLT2i are protective against stroke in people with T2D. Our RWE study demonstrated a protective effect of this class of drugs, compared to users of other antidiabetic drugs with T2D, which was similar to the conclusions reached in the meta‐analysis of randomized controlled trials (RCTs) by Kim et al. [9] but not by many other meta‐analyses [10]. Discrepancies between RCT and RWE studies can arise because of differences in experimental design, patient populations, unrecognized confounding factors, and adherence [19]. Of note, our results should still be interpreted with caution, given that *E*‐values ≤ 1.5 suggest the result of possible unobserved confounding. The *E*‐value represents the minimum strength of association, on the risk ratio scale, that an unmeasured confounder would need to have with both the treatment and outcome to fully explain away a specific treatment–outcome association. It reports how strongly an unmeasured confounder must be related to the treatment and outcome to explain away an effect estimate [20]. Confounding by indication occurs when the underlying condition for which a drug is prescribed influences both the likelihood of receiving the drug and the risk of experiencing a particular side effect. In studies involving drug use and side effects, confounding by indication can be a concern if the clinical indication for a drug also influences the outcome of interest [21]. For example, if an SGLT2i is prescribed more frequently to patients with features of diabetes that are already protective of stroke, then the observed association between use of this type of the drug and stroke might be due to the underlying protective condition, rather than the drug itself. In our study, as previously mentioned, some of the associations had *E*‐values below 1.5, indicating that only a small amount of unmeasured confounding would be needed to explain away these effects, and indicating weaker evidence for causality. We did not identify or test for a protective factor associated with greater use of SGLT2i to include in our PSM process (if this factor even exists). Our RWE might not have identified such a relationship among our cohorts had it been known because of potential limitations of our database.

However, some low *E*‐values in our study might lead to a needlessly pessimistic impression of the identified associations for two reasons. First, unmeasured factors in this study might be associated with strong confounders that were already accounted for in our PSM process, and second, it is unlikely that a confounding factor will be identified that influences both greater selection of SGLT2i and protection from stroke [22]. Without conducting a prospective trial and controlling for all confounders (including those that could influence both the choice of an SGLT2i, as well as the risk of stroke), then a retrospective RWE study like this one may be the best we can do to assess whether SGLT2is have a protective effect on stroke.

Rodriguez‐Valadez et al. [23] reported that the hazard ratio for stroke increased with increasing hematocrit concentrations, and they hypothesized that this increase in hematocrit was potentially an indirect effect of SGLT2i and that “limited data from SGLT2 inhibitor trials indicated loss of treatment effect for stroke with greater increase in hematocrit.” Our database did not contain enough propensity‐matched SGLT2i user and non‐SGLT2i user patients with robust pre‐ and post‐stroke hematologic data to address this hypothesis.

## 5. Limitations

We recognize a major limitation of our study, which is that the minimum follow‐up from the index date was set to be 365 days, meaning that we required subjects to survive for one year after the start of medication. Anyone who had a fatal stroke within the year post‐SGLT2i/other antidiabetic would be excluded. Although deaths are generally specified in a prospective study, in a retrospective study of hospital records like ours, data on deaths is not necessarily available if a patient were to die at home. This analysis could help determine the impact on different types of stroke outcomes compared to each other of SGLT2i. We also did not analyze stroke outcomes according to individual drugs within the SGLT2i class.

## 6. Conclusion

In conclusion, from both our RWE analysis and previous clinical trial data, it appears that SGLT2i are protective of stroke. By assessing this relationship, respectively, and comparing our results with previously collected prospective data, we have provided evidence to support this conclusion based on two different types of study designs.

**Nomenclature**
ATC: Anatomical Therapeutic Chemical; Common Data Model
AwT2D: adults with Type 2 diabetes
CI: confidence interval
COVID‐19: coronavirus disease of 2019
CPT: current procedural terminology
CV: cardiovascular
d: days
Dx: diagnosis
DPP‐4: dipeptidyl peptidase 4
hdPS: high‐dimensional propensity score
ICD‐9, ICD‐10: International Classification of Diseases 9th or 10th Revision Code
LASSO: least absolute shrinkage and selection operator
LOINC: Logical Observation Identifiers Names and Codes
NA: not applicable
OMOP: Observational Medical Outcomes Partnership
OR: odds ratio
PSM: propensity score matching
RCT: randomized clinical trial
RWE: real‐world evidence
Rx: medical prescription
SD: standard deviation
SGLT1: sodium‐glucose cotransporter 1
SGLT2: sodium‐glucose cotransporter 2
SGLT2i: sodium‐glucose cotransporter 2 inhibitors
TIA: transient ischemic attack
T2D: Type 2 diabetes
T2DM: Type 2 diabetes mellitus

## Author Contributions

Conceptualization and study design: David C. Klonoff and Saurabh Gombar. Data curation and investigation (recruitment, data collection, lab work, chart review): Agatha F. Scheideman, Mandy M. Shao, Alessandra T. Ayers, Cindy N. Ho, and Honor Magon. Formal analysis and statistics: Honor Magon, William C. Pike, Michael L﻿ Jackson, and Umesh Masharani. Supervision and project administration: David C. Klonoff.

## Funding

No funding was received for this manuscript.

## Disclosure

The authors have nothing to report.

## Conflicts of Interest

A.T.A. and C.N.H. are consultants for Liom. W.C.P. and S.G. are employees and stockholders of Atropos Health. H.M. is an employee of Atropos Health, and previous employee and stockholder of Komodo Health. M.L.J. is a former employee and current consultant for Atropos Health. D.C.K. is a consultant for Afon, embecta, Glucotrack, Lifecare, Novo, SynchNeuro, and Thirdwayv. A.F.S., U.M., and M.M.S. have nothing to disclose.

## Data Availability

The data that support the findings of this study are available from the corresponding author upon reasonable request.
